# Influence of Sequence Changes and Environment on Intrinsically Disordered Proteins

**DOI:** 10.1371/journal.pcbi.1000497

**Published:** 2009-09-04

**Authors:** Amrita Mohan, Vladimir N. Uversky, Predrag Radivojac

**Affiliations:** 1 School of Informatics and Computing, Indiana University, Bloomington, Indiana, United States of America; 2 Institute for Intrinsically Disordered Protein Research, Center for Computational Biology and Bioinformatics, Department of Biochemistry and Molecular Biology, Indiana University School of Medicine, Indianapolis, Indiana, United States of America; 3 Institute for Biological Instrumentation, Russian Academy of Sciences, Moscow Region, Russia; National Cancer Institute, United States of America and Tel Aviv University, Israel

## Abstract

Many large-scale studies on intrinsically disordered proteins are implicitly based on the structural models deposited in the Protein Data Bank. Yet, the static nature of deposited models supplies little insight into variation of protein structure and function under diverse cellular and environmental conditions. While the computational predictability of disordered regions provides practical evidence that disorder is an intrinsic property of proteins, the robustness of disordered regions to changes in sequence or environmental conditions has not been systematically studied. We analyzed intrinsically disordered regions in the same or similar proteins crystallized independently and studied their sensitivity to changes in protein sequence and parameters of crystallographic experiments. The observed changes in the existence, position, and length of disordered regions indicate that their appearance in X-ray structures dramatically depends on changes in amino acid sequence and peculiarities of the crystallographic experiment. Our study also raises general questions regarding protein evolution and the regulation of protein structure, dynamics, and function via variations in cellular and environmental conditions.

## Author Summary

Intrinsically disordered proteins, proteins that exist as conformational ensembles without time-invariant residue positions, have emerged as an important and common class of proteins in all kingdoms of life. Disordered proteins are characterized by distinct amino acid preferences, distinct mechanisms of binding, distinct substitution patterns and rates of evolution, and functional roles predominantly related to signaling and regulation. In recent years, disordered proteins have also been linked to human disease, both through conformational diseases or via host-pathogen interactions. However, despite increased importance, most studies of disordered proteins do not consider the environmental context in which the protein is found or the level of sequence change that would strongly influence the property of being disordered. To address this, we studied and quantified the variability of intrinsically disordered protein regions under different external conditions, such as temperature or pH, and compared them to the variability introduced by small sequence changes. We found that both have a strong impact on the existence of disordered regions, thus potentially regulating protein function by environmental factors or facilitating evolutionary change.

## Introduction

In the past decade, significant progress has been achieved in our understanding of the ubiquity and function of intrinsically disordered proteins [1]–[8]. What once seemed to be a set of exceptions to the traditional structure-to-function paradigm, where every protein was believed to have unique and stable 3D structure to carry out specific function, turned into a field where computational and experimental approaches were developed and combined to accurately characterize disordered proteins [9], understand their function [4],[7],[8] or mechanisms of binding [10]–[13], and estimate their abundance in the protein universe [14]–[16]. Undoubtedly, bioinformatics analyses and methods played a significant role in this process, especially a set of predictors and statistical techniques [8],[17]. However, despite previous success, questions can be raised about the generality of our view of disordered proteins in terms of sequence-to-structure determinants and influence of environmental conditions. Here, we attempt to address these questions by investigating the variability of observed disordered regions with changes in sequence and environmental conditions used for crystallization.

Recent studies document the effects of varying environmental conditions on regions of intrinsic disorder in similar proteins. Zurdo et al. studied two yeast ribosomal stalk proteins, P1α and P2β, which have different functional roles despite high sequence similarity and suggested that their functional differences stem from different structures [18]. Although neither protein is compact in solution and possesses folded structure under physiological pH and temperature, P1α was found to be mostly disordered with low helical content, whereas P2β had significant residual structure. This residual structure disappeared at temperatures below 30°C, but was regained under low pH or in the presence of trifluoroethanol. Palaninathan et al. reported that conformational changes were observed in the tertiary and quaternary structures in the crystals of the native human transthyretin (TTR) [19]. At pH = 4.0, TTR forms a tetramer and its crystal structure includes electron density for a functionally important EF helix-loop region. At pH = 3.5, this region is completely disordered.

**Figure 1:**
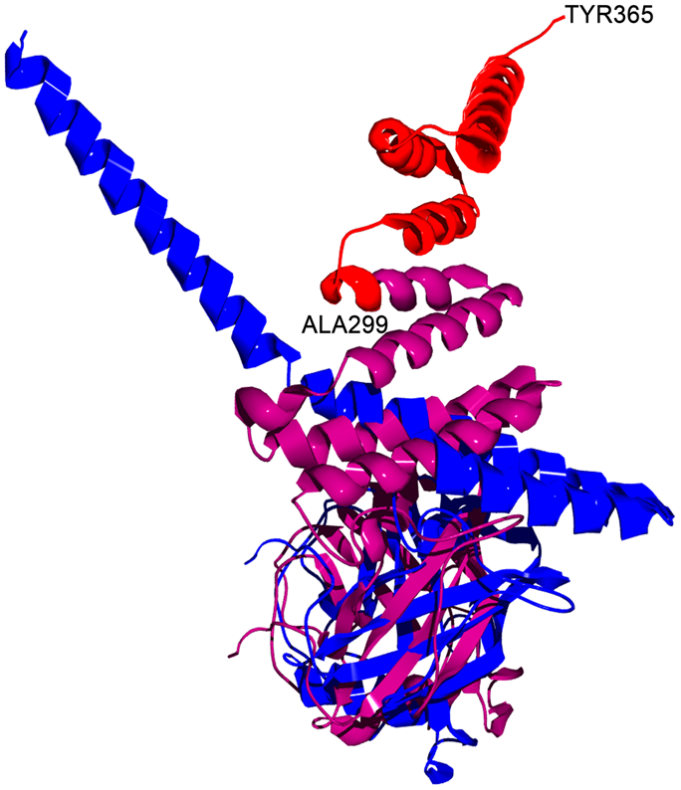
Structural alignment, using DALI, of two crystal structures of cyclophilin 40. Molecule 1IIP-A (blue) and 1IHG-A (pink) were crystallized under different pH values (8.0 vs. 6.1) and solved in different space groups. Regions that are observed as disordered in 1IHG-A are colored in red.

Our search of the Protein Data Bank (PDB) resulted in additional examples where slight changes in experimental conditions strongly correlated with the presence or absence of disordered regions. One such case is cyclophilin 40 (Cyp40), shown in Figure 1 (complete list of analyzed proteins can be found in Table S1, Suppl. Data). Cyp40 is one of the principal members of a family of large immunophilins found in mammals. The exact biological function of large immunophilins is incompletely understood, though they are believed to be strongly associated with Hsp90 and play a crucial regulatory role in the upkeep of steroid receptor activity. In PDB, Cyp40 is stored as 1IIP-A (tetragonal form) and 1IHG-A (monoclinic form). Both structures were obtained using the vapor diffusion, hanging drop method with recorded temperature of 277K, but 1IIP-A was crystallized at a pH of 8.0, whereas 1IHG-A was crystallized at pH of 6.1. The two proteins are identical, yet a rmsd of 14.2 Å was obtained from their structural alignment. Importantly, 1IHG-A contains an ordered region A299-Y365 that was absent from the structure of 1IIP-A (Figure 1). Neither protein was solved in the presence of natural ligands.

In addition to experimental studies, computational analyses of redundant sets of experimentally determined structures for identical protein regions have provided evidence of the existence of numerous protein fragments observed in both ordered and disordered states [20]. The authors analyzed these ‘dual-personality’ fragments and showed that they are characterized by amino acid compositions different than those for either ordered or disordered proteins and that their main functional roles are regulatory.

The examples discussed above demonstrate the strong influence experimental parameters can have on disordered residues in crystallized proteins. However, a hypothesis that variation in experimental conditions could potentially trigger structural changes affecting the existence, position or length of intrinsically disordered regions has not been systematically tested and quantified. In the following work, we provide evidence of significant variation of disordered regions, and protein structures in general, under the same or different experimental conditions that we believe can serve as a basic indicator of environmental regulation of protein structure and disordered regions *in vivo*.

## Results

To estimate the consistency of disordered residues and regions in protein crystal structures, we studied the overlap between disordered regions in pairs of highly similar proteins crystallized in independent experiments. At least one protein sequence in a pair was required to contain disordered regions of length≥3 residues and two proteins were considered similar if their global sequence identity was ≥90%. We investigated the influence of temperature, pH value, and salt concentration at the time of crystallization. To facilitate this analysis, each experimental factor was clustered into two groups, low and high (Materials and Methods). Thus, we refer to the experiments carried out under conditions clustered in the same or different groups as same (similar) and different (dissimilar) experimental conditions, respectively.

### Consistency of intrinsically disordered residues

**Figure 2:**
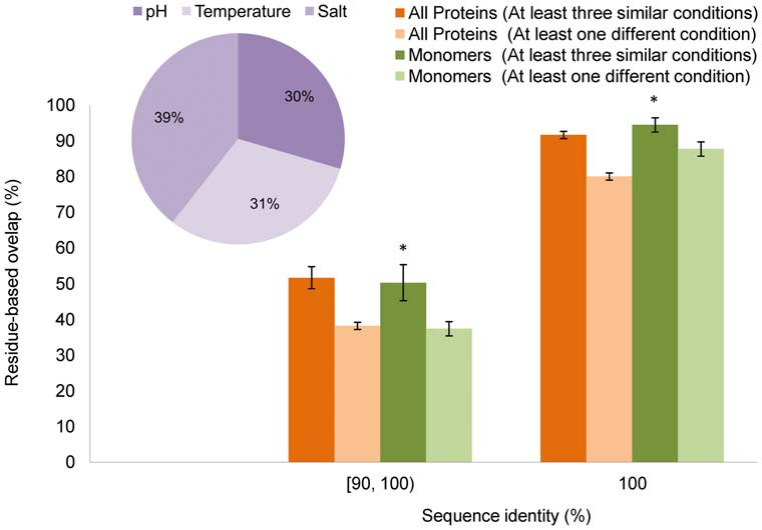
Percentage of overlap of disordered residues between protein pairs with sequence identity [90, 100)% and identical proteins. Set of all proteins is shown in orange and monomers are shown in green. Proteins were crystallized under at least one different experimental condition or three similar experimental conditions. Bars with asterisk (*) indicate results obtained using less than 100 proteins. (Inset) Percentage of times temperature, pH, and salt conditions changed when non-zero overlap occurred between a pair of proteins. P-values for 100% identity vs. [90, 100)% groups: all proteins with three similar conditions *P* = 4.0⋅10^−62^, all proteins with at least one different condition *P* = 1.8⋅10^−109^, monomers with three similar conditions *P* = 7.3⋅10^−11^, monomers with at least one different condition *P* = 3.5⋅10^−31^. P-values for 100% identity group: all proteins with three similar conditions vs. at least one different condition *P* = 1.6⋅10^−26^, all monomers with three similar conditions vs. monomers with at least one different condition *P* = 5.6⋅10^−3^. P-values for [90, 100)% identity group: all proteins with three similar conditions vs. at least one different condition *P* = 1.4⋅10^−3^, all monomers with three similar conditions vs. monomers with at least one different condition *P* = 3.4⋅10^−2^.

Figure 2 shows the mean agreement of disordered residues obtained in pairs of identical proteins and proteins with sequence identity in the range [90, 100)%. When all experimental conditions were similar, the agreement of disordered residues for identical sequences was 92% (95% for monomers only). For the same set of experimental conditions, however, and sequence identity in the range [90, 100)%, the agreement of disordered regions decreased to 52% for the set of all protein chains (*P* = 1.4⋅10^−48^; Wilcoxon test) and 50% for monomers (*P* = 5.5⋅10^−10^; Wilcoxon test). We also investigated the situation when at least one experimental condition was different (e.g. temperature, salt concentration, and/or pH value). For both identical proteins and those in the [90, 100)% range, the reduction of the mean agreement of residues designated as disordered was about 11 percentage points (see Fig. 2 caption for P-values). In an attempt to estimate which of the experimental conditions had the largest influence on the variability of observed disordered regions, a count for each condition was incremented for each protein pair with inexact matches of disordered regions whenever this condition differed. We found that salt concentration had slightly larger impact (39%) than temperature (31%) and pH value (30%), as shown in Figure 2 (inset). Furthermore, we found that, in general, an increase in temperature (6%) and pH value (7%) lead to an increase in the number of disordered residues in identical or similar protein chains. In contrast, an increase in salt concentration (11%) leads to a decrease in the number of observed disordered residues.

**Figure 3 pcbi-1000497-g003:**
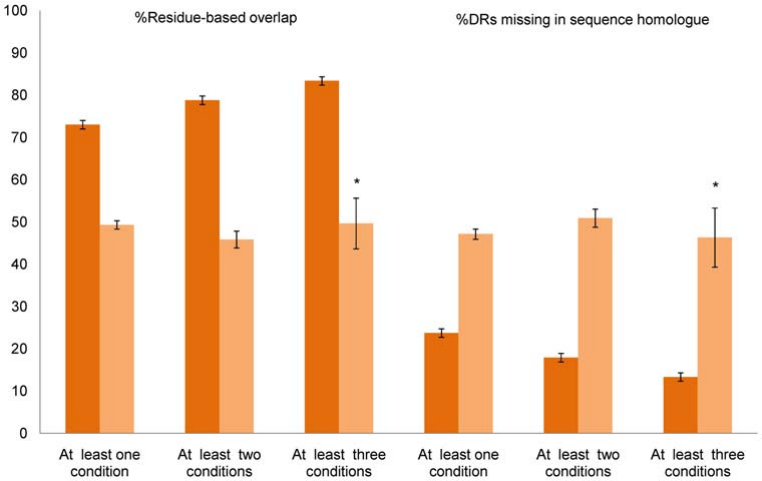
Consistency of disordered residues and regions as a function of experimental conditions. (Left) Percentage of overlap of disordered residues for pairs of proteins whose sequence identity is ≥90% and crystallized under at least one, two, and three similar and different experimental classes. (Right) Percentage of disordered regions that were observed as ordered in their entirety between the same set of protein pairs. Bars with asterisk (*) indicate results obtained using less than 100 proteins.

We also grouped all pairs of sequences with identity≥90% into those solved using at least one, two, or three similar experimental conditions and at least one, two, or three different experimental conditions. We estimate that, assuming unchanged experimental platforms for structure determination, the mean agreement of intrinsically disordered residues is 73% (79%, 83%) if one (two, three) or more experimental conditions are similar (Figure 3, left). When different experimental conditions were considered, the agreement of disordered residues was consistently around 50%.

In Table 1 we present complete results of the consistency measurements for both ordered and disordered regions for the pairs of chains with sequence identity≥90%. Ordered regions from such pairs of proteins appeared as highly overlapping (>98%), which is due to the unbalanced number of ordered and disordered residues in the non-redundant data set (14∶1 ratio).

**Table 1:** Mean overlap for disordered (D) and ordered (O) regions for protein pairs with ≥90% sequence identity crystallized under similar and different experimental conditions.

		At least one condition	At least two conditions	At least three conditions
**Same**	Number of proteins	4086	3488	852
**Conditions**	Mean D overlap	73.0	78.8	83.4
	Mean O overlap	98.7	99.0	99.1
	Mean accuracy	85.9	88.9	91.2
	DRs missing (%)	23.8	17.9	13.3
**Different**	Number of proteins	1427	440	42
**Conditions**	Mean D overlap	49.3	45.9	49.7
	Mean O overlap	98.0	98.4	98.8
	Mean accuracy	73.7	72.1	74.2
	DRs missing (%)	47.2	50.9	46.3

Mean accuracy is an average of overlaps between ordered and disordered regions.

**Figure 4:**
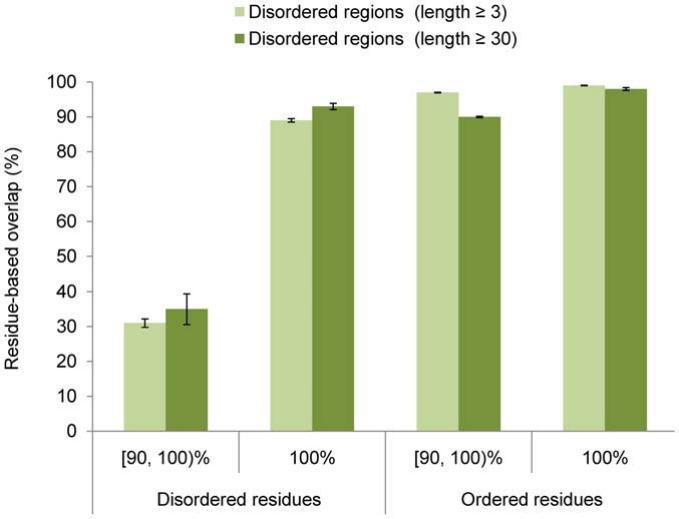
The mean observed agreement between ordered and disordered residues in similar and identical protein chains. All disordered regions (≥3 residues) and long disordered regions (≥30 residues) are separately presented. The P-values for the [90, 100)% and 100% sequence identity pairs were *P* = 0.29 and *P* = 0.02, respectively.

Finally, we estimated the mean agreement of disordered residues using pairs of similar and identical protein sequences wherein experimental information at the time of pair generation was not considered. If identical protein pairs are considered, the mean overlap of disordered and ordered residues was 89% and 99%, respectively. When we considered disordered regions of length 30 or more, the mean overlap was 93% and 98%, respectively (Figure 4). Interestingly, all pairs from our analysis in which long disordered regions significantly differed belonged to dissimilar experimental classes thus strongly suggesting that the appearance of disordered regions is influenced by variations in experimental conditions (e.g. 1COT-B and 1S6P-B). Consideration of similar sequences resulted in a significant reduction in the mean overlap: 31% for all disordered regions and 35% for long disordered regions only. Note that the slightly smaller overlap of disordered residues, compared to the one from Figure 2, is due to the influence of completely ordered proteins for which we were unable to extract experimental conditions and therefore were excluded from the analysis in Figure 2.

### Consistency of intrinsically disordered regions

The observed consistency of disordered residues may not necessarily be the same at the level of entire disordered regions. Figure 3 (right) shows the percentage of disordered regions that were found as ordered in their entirety when the same or similar proteins were crystallized in independent experiments. When all crystallographic parameters were similar, 13% of regions were found as completely ordered. On the other hand, when all parameters were different we estimated that close to 50% of the regions were lost (*P* = 1.7⋅10^−10^; Wilcoxon test).

To understand whether a loss of disordered regions could be due to potential ligand binding, we investigated pairs of proteins (*p*_1_, *p*_2_), where *p*_1_ contained a disordered region *r* for which *p*_2_ contained all ordered residues in the segment aligned with *r*. We considered that a ligand influenced disorder-to-order transition if any of its atoms could be found within 10 Å of any of the ordered residues from *p*_2_ corresponding to *r* as well as requiring that the ligand was not present in the model of protein *p*_1_. We found that about 25% of disordered regions that underwent order-disorder transition were due to direct ligand binding. Thus, ligands in PDB considerably influence the existence of disordered regions. However, their influence appears to be a less significant factor than experimental conditions or sequence variation.

### Predictability of intrinsically disordered residues

The results presented in Table 1 and Figures 2–4 provide estimates regarding the limits of predictability of intrinsically disordered residues. By combining the mean agreement of both ordered and disordered residues in identical protein chains when all experimental conditions agree, we estimate that the prediction accuracy of computational models constructed to predict disordered regions, measured by averaging sensitivity and specificity, is approximately 95%. This accuracy reduces to 90% if the experimental conditions are not taken into consideration, which is closer to the situation used in computational studies. However, since we considered only identical pairs of proteins, both of these limits seem overly optimistic. Thus, we believe that a more realistic estimate is provided when all sequence pairs with identity≥90% are considered and experimental conditions are ignored. The observed agreement of disordered and ordered residues in such a case was 66% and 96%, respectively. Thus, the maximum balanced-sample accuracy is probably about 81%. Interestingly, the best models in CASP7 assessment have reached 74–78% balanced-sample accuracy [21], so it is unclear whether the current general predictors can be significantly improved. The knowledge of experimental conditions, on the other hand, should be able to improve the predictability of disordered residues by at least 5 percentage points (Figure 2). In addition, structures of solved homologs and mutants could provide an additional increase if the points of low stability can be identified.

## Discussion

This study addresses the relationship between intrinsically disordered protein regions, protein sequence, and parameters of crystallographic structure determination. The existence, position, and length of disordered regions in highly similar proteins was shown to strongly depend on variation in amino acid sequence as well as the parameters of crystallographic experiments, such as temperature, pH, and salt concentration. For identical protein chains, most of the observed rearrangements in the crystal lattice can be explained by variation in experimental conditions. For highly similar chains, both experimental conditions and the intrinsic change of protein structure were significant factors. However, we are hesitant to assign relative importance to these factors since the observed sequence differences in PDB are likely to be non-random (for example, mutations with functional or phenotypic significance are frequently of interest for structure determination). The presence/absence of ligands appeared to be less significant in our analysis.

**Figure 5:**
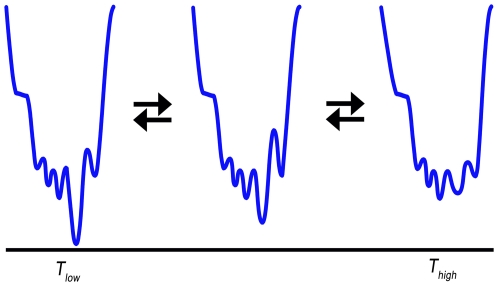
Stylized depiction of the energy landscape as a function of the environment. For low temperatures, on the left, the energy landscape is characterized by a dominant, high-probability, conformation (ordered state). For high temperatures, on the right, the valley of the landscape flattened and became rugged without any single dominant conformation (disordered state).

The presence of a disordered region under one set of experimental conditions and absence under another can be understood through the framework of the probabilistic theory of protein folding. At every time instant, a protein can be assigned a probability of any particular conformation based on its energy landscape [22],[23]. For ordered proteins, such energy landscapes are characterized by single (or a small number of) deep minima with high probabilities associated with the corresponding conformations. Since the number of conformations in the high energy states is huge and the barriers for moving away from the dominant conformation are relatively large, the energy landscape has a shape of a funnel [23]. This minimum energy state is often associated with protein function and is called the native state. On the other hand, the energy landscapes for disordered proteins are shallower, typically characterized by flat and rugged valleys, i.e. they contain a large number of energy minima with relatively small barriers for transitioning between distinct conformations [24]. Consequently, the probability of each conformation corresponding to an energy minimum is relatively low. The absence of a high probability conformation eventually leads to missing electron density during crystallographic experiments. Thus, the variability in structures of identical proteins solved under different experimental conditions is caused by the environment-driven changes of the energy landscape (Figure 5). The altered probability distribution over the space of allowed tertiary structures ultimately results in a population shift between ensembles of pre-existing conformational isomers [23]–[25].

The folding funnel theory can not only accommodate both the thermodynamic and the kinetic requirements for protein folding [22], but also provide a general framework under which folding, binding (including allostery), or effects of mutations and post-translational modifications can be considered [23],[24],[26]. For example, folding and binding essentially represent the same phenomenon with a distinction that the chains are disconnected in the case of binding [23],[25],[27]. In allostery, a lower probability conformation may be the one preferred for binding. If this complex is the preferred state, the increased probability of a bound conformation will cause a population shift over time from one dominant conformation to the one preferred for binding [26],[28]. Recently, population shifts were demonstrated for ubiquitin, where all bound conformations available from crystallographic experiments were shown to be accessible in solution by NMR [29].

A limitation of our analysis is that it only included disordered proteins with at least two deposited structures in PDB, and thus may be a non-representative sample. In addition, this data set is enriched for short disordered regions that have distinct sequence biases relative to long regions [30],[31]. A full analysis including long disordered regions was not possible due to the small number of available protein pairs; however, the overall trends indicate that long disordered regions may be equally sensitive to variation in sequence and experimental conditions.

In general, this work provides evidence that disordered protein regions are very sensitive to changes in amino acid sequence and experimental conditions of crystallographic experiments. The success of such crystallographic experiments depends on the complexity of protein's structure and also on a number of experimental or environmental factors including purity of the protein sample, temperature, ionic strength, pH, and precipitants such as ammonium sulfate or polyethylene glycol [32]. Undoubtedly, there are a number of factors that distinguish crystallization conditions from physiological conditions, but there is also a body of evidence that protein structures often correspond to their native states [32]. Therefore, it is reasonable to speculate that a wide range of intracellular and extracellular conditions may have similar effects on the dynamics of protein 3D structure *in vivo*. The habitats for many living organisms vary from acidic to cold or hot, with various species being able to tolerate wide ranges of environmental conditions. As suggested and quantified by our analysis, any similar variations in cellular environments could have profound effects on protein structure, dynamics, and function. Sensitivity to sequence changes, on the other hand, may facilitate the evolution of function, especially for proteins with the same fold classification.

## Materials and Methods

### Data sets

Our initial data set *S* comprised of 18,884 protein chains from PDB (March 2008) characterized by X-ray crystallography with resolution of at most 2 Å (Table S2, Suppl. Data). It contained two subsets: *D*–a set of 14,646 chains containing at least one disordered region of length≥3, identified as those missing C-α atoms in the ATOM fields; and *O_D_*–a set of 4,238 completely ordered chains such that each sequence was ≥90% identical to one or more sequences in *D*. For each sequence in *S* we extracted experimental conditions: temperature, pH value, and concentration of salt (e.g. ammonium sulfate, potassium sodium tartrate, sodium cacodylate, and a number of others), whenever available (1 sequence in *D* and 1502 sequences in *O_D_*, did not have any experimental conditions extracted due to differences in file format). While temperature and pH value can be obtained from designated fields in PDB, the salt concentration was mined from REMARK200 and REMARK280 fields and manually checked in a number of cases. For simplicity of our analysis, each experimental condition was clustered into two groups, *high* and *low*, as discussed in the Results section (Figure S1, Suppl. Data). Temperature was clustered into group high (*T_h_*), containing temperatures greater than or equal to 200 K and group low (*T_l_*), containing temperatures below 200 K at the time of experiment. pH value was clustered into *P_h_* and *P_l_* based on threshold 6.5, while the salt concentration was clustered into *S_h_* and *S_l_* based on the threshold of 100 mM.

To construct the non-redundant data sets, the initial set *D* was split into overlapping subsets, where each subset set *D_i_* contained proteins crystallized at experimental conditions *E_i_* ∈ {*T_h_*, *T_l_*, *T_h_P_h_*, *T_h_P_l_*, …, *T_l_P_l_S_l_*}. More specifically, data set containing proteins crystallized at conditions *T_h_P_h_*, had proteins solved at high temperature and high pH value, but the salt concentration could be from the entire range or unknown. Each data set *D_i_* was also filtered into a non-redundant set *D_i−nr_* such that no two chains had sequence identity greater than or equal to 25% on a global level (BLOSUM62 matrix, gap opening penalty = *−*11, and gap extension penalty = *−*1). This approach of defining non-redundant sets was used for estimating the overlap of disordered regions between classes *E_i_* and *E_j_*. The size of each data set is shown in Table 2.

**Table 2:** Number of proteins with available temperature, salt, and pH value data (pre- and post-removal of redundant proteins) along with respective number of disordered and ordered residues in each class.

		Temperature		Salt		pH	
		T_high_	T_low_	S_high_	S_low_	P_high_	P_low_
*D*	# proteins	3,675	14,822	4,413	1,986	11,715	6,136
	# disordered residues	41,868	220,068	55,870	24,191	158,063	96,378
	# ordered residues	788,496	3,150,810	831,521	393,542	2,534,009	1,306,568
*D_nr_*	# proteins	556	1,600	700	392	1,393	846
	# disordered residues	10,196	33,815	13,699	7,724	27,717	18,695
	# ordered residues	161,864	455,274	188,698	106,142	401,679	232,957

Only proteins with explicitly state values corresponding to temperature, pH or salt were used.

### Consistency of disordered residues and regions

Consistency of disordered residues and regions was estimated by calculating the mean overlap of ordered and disordered regions in similar or identical protein chains, crystallized at the same or different experimental conditions. Two protein chains were considered to be similar if their global sequence identity was ≥90%. This threshold was selected to ensure not only similar 3-D structure between two proteins [33], but also similar function [34].

**Figure 6:**
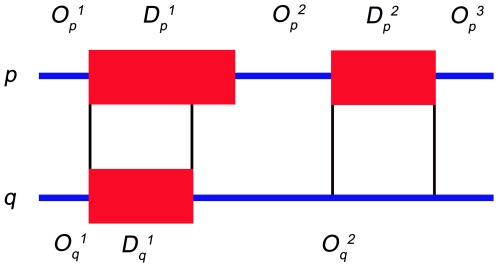
Calculation of the mean overlap between ordered and disordered residues between two homologous proteins *p* and *q*. About 30% of the disordered residues in *p* are disordered in *q* (the length of 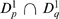 over the length of 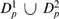). Similarly, 100% of disordered residues in *q* are disordered in *p* (the length of 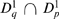 over the length of 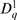). Thus, the mean agreement of disordered residues between *p* and *q* is about 65%. The mean agreement of ordered residues is calculated using the same approach.

The mean overlap between two globally aligned proteins *p* ∈ *D_i–nr_* and *q* ∈ *S_j_*, where the sequence identity (*si*) between *p* and *q* was greater than or equal to threshold *t*_1_ and lower than *t*_2_, was calculated as follows. Let *O_p_* and *D_p_* be the sets of positions of ordered and disordered residues in protein *p*, and *O_q_* and *D_q_* sets of positions of ordered and disordered residues in protein *q*, respectively, as shown in Figure 6. The residue positions are calculated after the alignments are completed. The indices corresponding to insertions and deletions, as well as the indices corresponding to disordered regions of length below 3, were ignored.

We calculate the overlap between ordered (*o_o_*) and disordered regions (*o_d_*) as
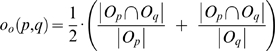


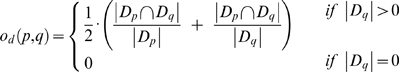



Note that *q* can be a completely ordered sequence, while *p* is guaranteed to contain at least one disordered region. The average overlap of ordered and disordered regions for a pair (*p*, *q*) is calculated as
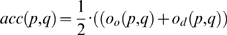



We use the term accuracy for the mean overlap due to its similarity to a prediction process in which ordered and disordered regions in one protein serve as predictions for the other protein.

The overlaps between pairs of proteins are then generalized to the level of data sets. An average accuracy for chain *p* is first calculated over all sequences *q* that are within the sequence identity range [*t*_1_, *t*_2_) from *p*, denoted by *si*(*p*, *q*) ∈ [*t*_1_, *t*_2_). Then, the average accuracy between data sets *D_i–nr_* and *S_j_*, corresponding to experimental conditions *E_i_* and *E_j_*, is calculated as the mean over all proteins *p*. We formalize the entire calculation as
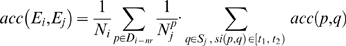
where 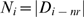 and 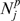 is the number of sequences *q* ∈ *S_j_* that when aligned to *p* have sequence identity in range [*t*_1_, *t*_2_). Assuming that the maximum prediction accuracy of intrinsically disordered regions is limited by an empirically observed agreement in similar proteins, this approach provides an estimate of the upper limit of the balanced sample accuracy over the given two sets of experimental conditions. The results for several groups of experimental conditions were obtained by simple group averages. The number of pairs for each group of experimental conditions is listed in Table S3 (Suppl. Data).

To quantify the agreement of disordered regions for two sets of experimental conditions *E_i_* and *E_j_*, we used a conceptually similar approach. For each protein *p* ∈ *D_i–nr_* we calculated the fraction of regions for which the overlap with sequence *q* ∈ *S_j_* was zero. The fraction of such regions in *p* was then averaged over all proteins from *q* ∈ *S_j_* where *si*(*p*, *q*) ∈ [*t*_1_, *t*_2_). Finally, the fraction of regions that undergo order-disorder transition between two sets of experimental conditions *E_i_* and *E_j_* was further averaged over all proteins *p* ∈ *D_i–nr_*.

Statistical confidence for the estimates was calculated by bootstrapping the non-redundant data sets *D_i–nr_* 10,000 times.

## Supporting Information

Figure S1 Histogram of observed temperature (a), pH (b), and salt concentration (c) in the data set.
(3.54 MB TIF)

Table S1 Complete list of analyzed protein pairs.
(0.12 MB XLSX)

Table S2 Complete list of analyzed proteins.
(0.59 MB XLSX)

Table S3 The number of pairs for each group of experimental conditions.
(0.02 MB XLSX)
