# Supplementary figures and images for: Influence of Sequence Changes and Environment on Intrinsically Disordered Proteins
Source: PLoS Comput Biol. 2009 Sep 4;5(9):e1000497. doi: 10.1371/journal.pcbi.1000497 (PMC2727479; doi:10.1371/journal.pcbi.1000497)

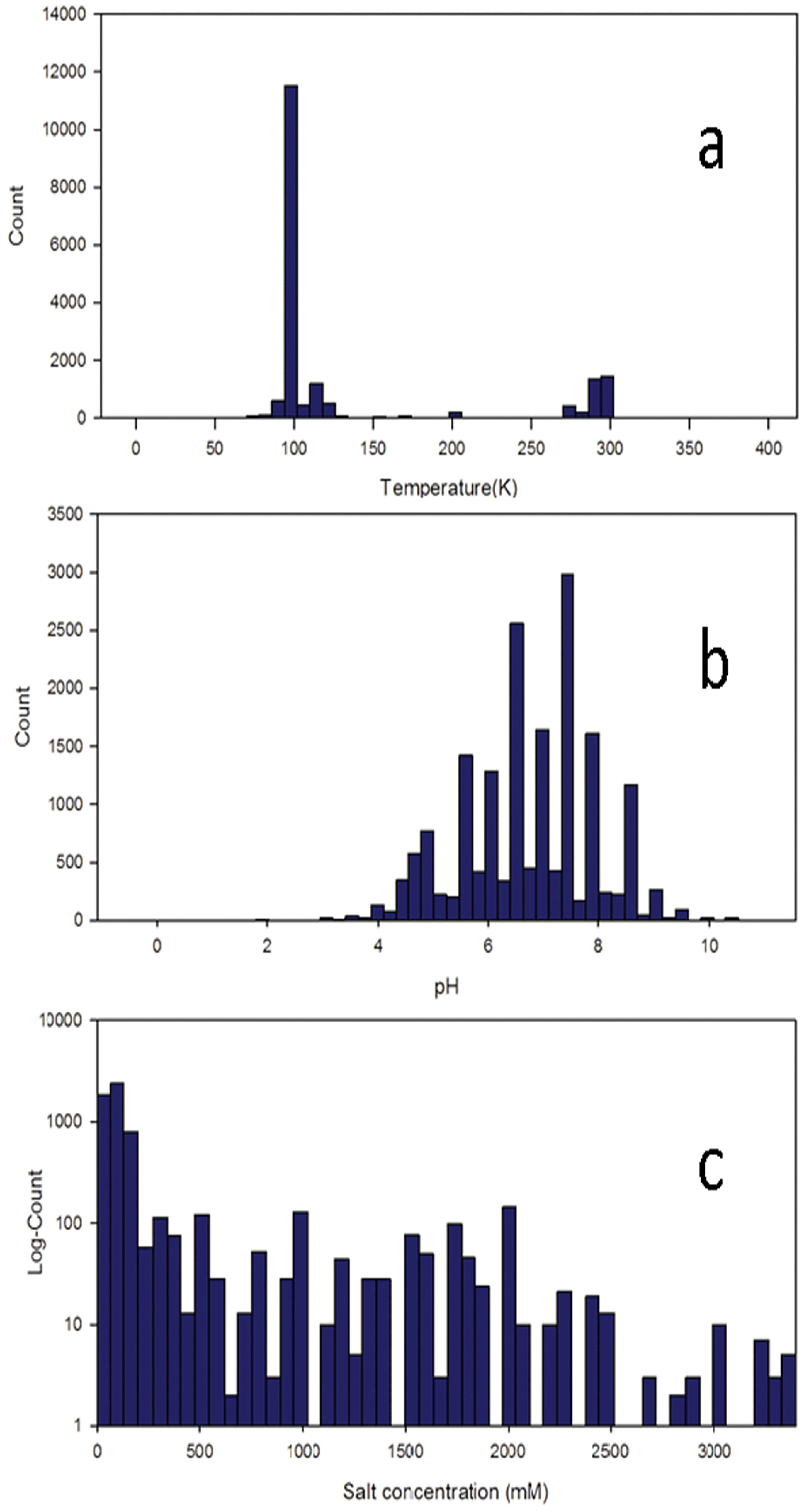

Supplement: Figure S1 — Histogram of observed temperature (a), pH (b), and salt concentration (c) in the data set. (3.54 MB TIF) [file pcbi.1000497.s001.tif]
